# Disparities in healthcare expenditures according to economic status in cancer patients undergoing end-of-life care

**DOI:** 10.1186/s12885-022-09373-y

**Published:** 2022-03-22

**Authors:** Kyu-Tae Han, Woorim Kim, Seungju Kim

**Affiliations:** 1 Division of Cancer Control & Policy, National Cancer Control Institute, National Cancer Center, Goyang, Republic of Korea; 2 Department of Nursing, College of Nursing, The Catholic University of Korea, 222, Banpo-daero, Seocho-gu, Seoul 06591 Seongnam, Republic of Korea

**Keywords:** Healthcare expenditure, End-of-life, Healthcare disparities, Cancer patients, Economic status

## Abstract

**Backgrounds:**

A desire for better outcome influences cancer patients’ willingness to pay. Whilst cancer-related costs are known to have a u-shaped distribution, the actual level of healthcare utilized by patients may vary depending on income and ability to pay. This study examined patterns of healthcare expenditures in the last year of life in patients with gastric, colorectal, lung, and liver cancer and analyzed whether differences exist in the level of end-of-life costs for cancer care according to economic status.

**Methods:**

This study is a retrospective cohort study which used data from the Korean National Elderly Sampled Cohort, 2002 to 2015. End-of-life was defined as 1 year before death. Economic status was classified into three categorical variables according to the level of insurance premium (quantiles). The relationship between the dependent and independent variables were analyzed using multiple gamma regression based on the generalized estimated equation (GEE) model.

**Results:**

This study included 3083 cancer patients, in which total healthcare expenditure was highest in the high-income group. End-of-life costs increased the most in the last 3 months of life. Compared to individuals in the ‘middle’ economic status group, those in the ‘high’ economic status group (RR 1.095, 95% CI 1.044–1.149) were likely to spend higher amounts. The percentage of individuals visiting a general hospital was highest in the ‘high’ economic status group, followed by the ‘middle’ and ‘low’ economic status groups.

**Conclusion:**

Healthcare costs for cancer care increased at end-of-life in Korea. Patients of higher economic status tended to spender higher amounts of end-of-life costs for cancer care. Further in-depth studies are needed considering that end-of-life medical costs constitute a large proportion of overall expenditures. This study offers insight by showing that expenditures for cancer care tend to increase noticeably in the last 3 months of life and that differences exist in the amount spent according economic status.

**Supplementary Information:**

The online version contains supplementary material available at 10.1186/s12885-022-09373-y.

## Introduction

Cancer exerts a noticeable cost burden worldwide. According to statistics published by the Organization for Economic Cooperation and Development (OECD), cancer treatment accounts for 3 ~ 7% of total health expenditure in OECD countries [1]. Studies have shown that cancer patients in the United States spend $16,346 per capita on health care per year, which is nearly four times higher than that spent by patients without cancer; in the case of European Union countries, cancer patients report spending an average of €102 per year on cancer care [2, 3]. The economic burden of cancer is also vast in Korea, which have nearly doubled over the past 10 years [4].

The cost of cancer treatment may vary depending on cancer type and stage of diagnosis, but characteristically, healthcare expenditures from diagnosis to death often show a U-shaped distribution [5]. Studies report that expenditures often upsurge at diagnosis, reduces and plateaus, and then again escalates at end-of life, which ultimately leads to patients spending the most at end-of-life [6, 7]. Previous literature focusing on Korea have shown that over 50% of total cancer costs were spent at the last year of life, with expenditures spiking in the last months [8]. Most of these costs are related to inpatient care, mostly because unlike many other countries, there is a general deficiency of facilities for treatment of terminal cancer and also because most available facilities hospitalization based [8]. However, studies on patterns of end-of-life expenditures in Korean cancer patients are lacking, in addition to whether differences exist based on income level.

Many epidemiological literature have reported the effect of socioeconomic status on health, suggesting that the lower economic groups generally practice healthy behaviors less and face difficulties in achieving health-related goals [9]. As health literacy mediates health status, especially in the lower economic status groups [10], poor health literacy may be associated with less medical knowledge, less use of preventive services, and increased health care costs [11, 12]. At the same time, an individual’s economic status can directly relate to the level of medical expenses spent, suggesting that medical expenses may vary depending on income level. A study conducted in the United States revealed that the pattern of medical spending according to economic status varied somewhat over time [13]. Specifically, the low-income group reported worse health and spending a high level of healthcare expenditures, but from 2004, the high-income group showed an increase in healthcare expenditures, which may reflect in willingness to pay or ability to pay [13].

Considering such tendencies, income level may affect a patient’s level of healthcare expenditures. Cancer treatment can directly affect patient outcomes, in which active treatment can increase the likelihood of survival [14]. Desire for better outcomes can impact willingness to pay in cancer patients, which is important because in addition to survival, quality of life is emerging as an important value in cancer care [15]. As a desire for a better life can affect willingness to pay for cancer screening and treatment, differences in healthcare expenditures can lead to a difference in outcomes according to income level [16, 17]. In particular, since the healthcare system of Korea has a weak referral system and patients can freely visit medical institutions, cancer patients of different income groups are likely to exhibit different patterns in end-of-life care seeking behaviors.

The purpose of this study was to investigate patterns of end-of-life healthcare expenditures in patients diagnosed with gastric, colorectal, liver, and lung cancer and to examine whether differences exist in the amount spent depending on the economic status of patients. End-of-life was defined as 1 year prior to death, in which further analysis was conducted by stratifying the time period into 12 to 4 months and 3 to 0 months before death. The hypothesis was that lower income patients would spend less than higher income patients. Possible reasons for such differences were deducted by further analyzing the type of healthcare institution patients of different economic status visited the most at end-of-life.

## Methods

### Healthcare system in Korea

The healthcare system of Korea operates as a single payment system and the entire population is covered by the National Health Insurance (NHI). Patients can visit a hospital of their choice relatively freely. The level of co-payment for outpatient care varies depending on the type of hospital but patient spending for reimbursed services does not exceed 20% of total costs for hospitalization. In the case of cancer, because it is recognized as a disease of high burden, the level of co-payment is 5% of total costs for both outpatient and inpatient care. The level of copayment is 0 ~ 5% of total costs for Medical Aid beneficiaries (See Supplementary Fig. a).

### Data and study population

This study used data from the Korean National Elderly Sampled Cohort collected based on the NHI. Information on a sample of around 10% (about 550,000 individuals) of the entire Korean population aged 60 years or above in 2002 were collected [18]. Information on demographic and socioeconomic characteristics, healthcare utilization and treatment, medical check-ups, and medical institution were included. The association between levels of healthcare expenditures at end-of-life and economic status was investigated in patients with gastric (C16), colorectal (C18-C20), lung (C33-C34), and liver (C22) cancer. These cancer types were selected as they are the most common types of cancer found in Korea. Diagnosis was based on the International Classification of Diseases, 10th revision (ICD-10).

To encompass only newly diagnosed patients, a wash out period of 5 years was applied. Patients who were newly diagnosed with any of the four types of cancer described above and who died between 2007 to 2015 were observed. Based on the analysis on medical expenditures at end-of-life (365 days before death), patients who died within 1 year from first diagnosis or patients who received treatment after 6 years from time of first diagnosis were excluded. Medical Aid beneficiaries were also excluded as they are subject to different levels of copayment and exhibit different characteristics compared to NHI covered individuals. Around 97% of the entire population are NHI beneficiaries in Korea. The final study population consisted 3083 individuals, which included 863 gastric, 898 colorectal, 882 lung, and 440 liver cancer patients.

### Variables

The outcome variable was healthcare expenditures at end-of-life, calculated based on the sum of inpatient and outpatient costs during the last 12 months of life. To adjust for the escalations in healthcare costs over time, a discount rate was applied based on the annual NHIS rate of increase. Additional analysis was conducted by stratifying healthcare expenditures into 12 to 3 months before death and 3 months to death to compare whether differences exist depending on the phase of end-of-life care.

The interesting variable was economic status, calculated based on the level of NHI premium paid by the study participants. NHI beneficiaries are divided into the employed (employees and employers) and self-employed groups, in which coverage is extended to all household members. NHI premiums are calculated based on income, property, and living standards, making it an economic indicator. Economic status was defined by the level of insurance premium paid by an individual, classified into the ‘low,’ (~ 30 percentile), ‘middle,’ (31 to 70 percentile), and ‘high’ (71+ percentile) groups.

Other independent variables included in this study were sex (male, female), age (~ 69, 70 to 74, 75 to 79, or 80+ years), type of insurance coverage (NHI employees or self-employed), cancer type, survival time after first diagnosis (1 to 2, 2 to 3, 3 to 4, 4 to 5, or 5 to 6 years), Charlson Comorbidity Index (CCI), residing area (capital area, metropolitan, or others), main treatment institution (general hospital, hospital, long-term care hospital, and others), sum of length of stay (LOS), and year. Charlson Comorbidity Index was incorporated to adjust for clinical severity, calculated based on records of medical symptoms in the last year of life. Symptoms related to cancer were excluded. Main treatment institution was classified based on the type of institution each patient spent the most in terms of healthcare expenditures.

### Statistical analysis

Overall monthly changes in total healthcare costs during the last year of life for each economic status group were calculated. The distribution and general characteristics of the study population were measured using analysis of variance (ANOVA). Multiple gamma regression analysis using the generalized estimated equation (GEE) model were conducted after controlling for all independent variables to investigate the association between healthcare costs at end-of-life and economic status. Additional analyses on the gamma regression were conducted by stratifying end-of-life costs into 12 to 3 months before death and 3 months to death. A comparison was also made between economic status groups based on the type of most commonly visited healthcare institution (general hospitals, hospitals, long-term care hospitals, or clinics). All statistical analyses were performed using the SAS statistical software version 9.4 (Cary, NC).

## Results

**Fig. 1:**
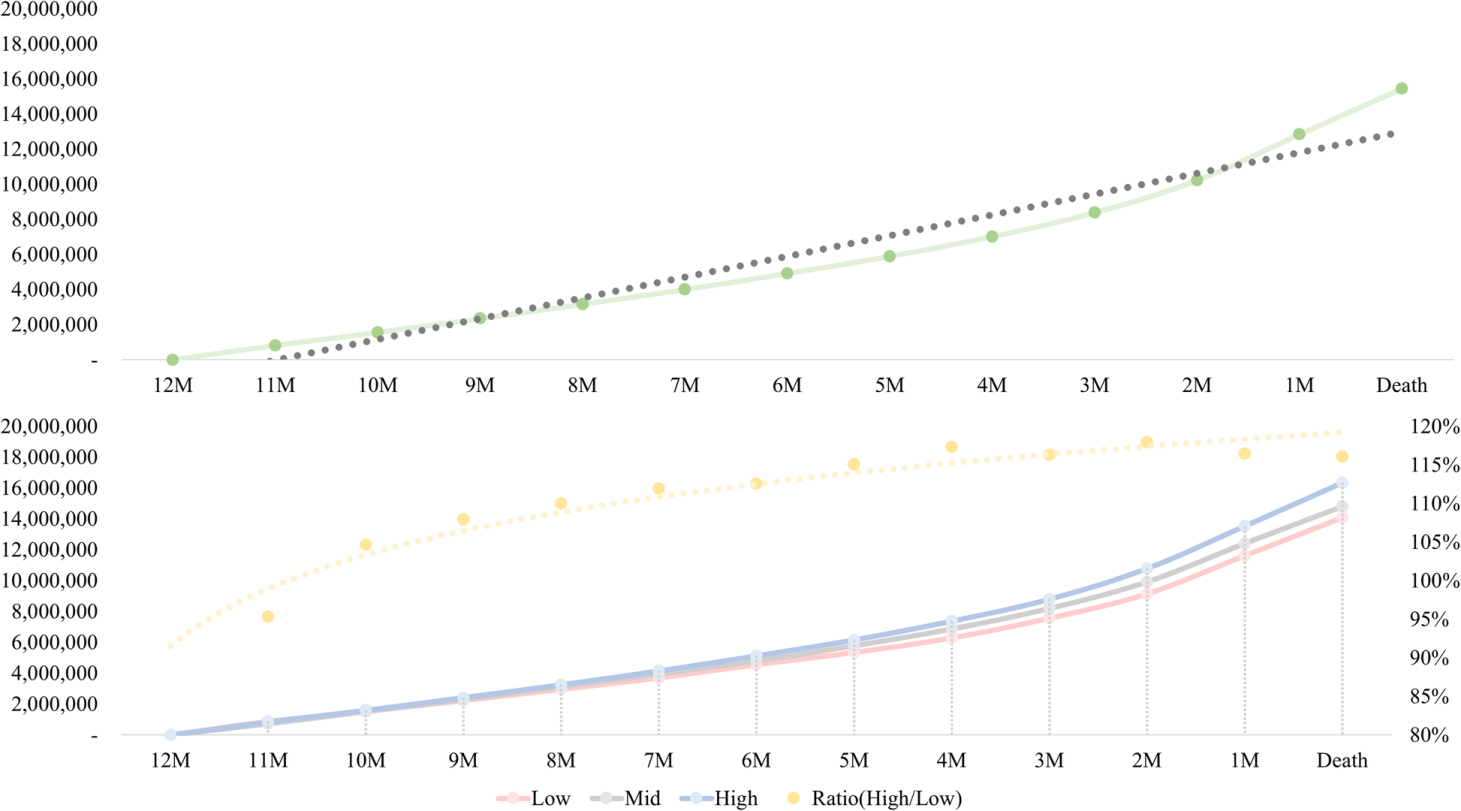
Average level of monthly healthcare expenditure at end-of-life and differences in expenditure according to economic status

Figure 1 shows the trend of cumulative healthcare expenditure at end-of-life, in addition to the differences in ratio between the ‘high’ and ‘low’ economic status groups. End-of-life costs increased the most in the last 3 months before death. The ratio between the two groups tended to fluctuate between 110 and 120% and escalated slightly as participants neared end-of-life, as depicted in Fig. 1. The average amount of end-of-life healthcare expenditures spent by the study participants according to economic status are shown in Table 1. The average end-of-life healthcare expenditure was 15,500,000 Korean Won (KRW) (1 USD = approximately 1135 KRW). Total end-of-life expenditures were highest in the ‘high’ economic status group (Mean 16,300,000 KRW, SD 15,100,000), followed by the ‘middle’ (Mean 14,800,000 KRW, SD 12,700,000) and ‘low (Mean 14,000,000, SD 12,800,000) groups. Similar tendencies were found when examining healthcare expenditures separately for 12 to 3 months and 3 to 0 months prior to death.

**Table 1:** Average end-of-life healthcare expenditures spent by the study participants

						Unit: 1,000,000 KRW	
**Variables**	**N**	**Healthcare expenditures**					
		**Total**		**12 ~ 3 Months**		**3 Months ~ Death**	
		**Mean**	**SD**	**Mean**	**SD**	**Mean**	**SD**
**Economic status**							
Low	578	14.0	12.8	7.6	8.9	6.5	7.5
Mid	900	14.8	12.7	8.2	9.2	6.6	6.7
High	1605	16.3	15.1	8.8	10.6	7.5	7.5
**Sex**							
Male	1973	15.7	14.1	8.5	9.8	7.2	7.6
Female	1110	15.0	13.9	8.2	10.1	6.8	6.6
**Age**							
~ 69	157	21.1	14.3	12.5	10.0	8.6	7.4
70 ~ 74	907	18.6	13.8	10.6	10.4	8.0	7.0
75 ~ 79	941	15.9	16.3	8.6	11.5	7.3	8.0
80~	1078	11.6	10.7	5.7	7.0	5.8	6.6
**Type of insurance coverage**^**a**^							
NHI (self-employed)	938	15.9	14.5	8.9	10.3	7.0	7.2
NHI (employees)	2145	15.3	13.8	8.2	9.8	7.0	7.3
**Types of cancer which diagnosed**							
Gastric cancer	863	14.2	13.9	7.9	9.5	7.3	7.9
Colorectal cancer	898	15.0	15.5	7.6	10.9	6.4	7.3
Hepatocellular carcinoma	440	16.7	12.9	8.7	8.9	8.0	7.0
Lung cancer	882	16.6	13.1	9.5	9.8	7.1	6.7
**Survival time (from first diagnosis)**							
1y ~ 2y	877	15.1	12.6	8.4	8.5	6.7	7.1
2y ~ 3y	1037	14.8	12.6	8.1	9.3	6.6	6.3
3y ~ 4y	590	15.2	13.3	8.1	10.0	7.1	6.6
4y ~ 5y	368	17.6	18.7	9.6	13.4	8.0	8.7
5y+	211	17.4	18.1	8.3	11.4	9.1	10.4
**Charlson Comorbidity Index**							
~ 2	541	8.6	9.2	4.5	6.4	4.2	5.6
3 ~ 5	639	11.8	10.5	5.4	6.6	6.3	7.2
6~	1903	18.6	15.2	10.5	11.0	8.1	7.4
**Residing area**							
Capital area	1055	16.8	15.0	9.1	10.3	7.6	8.2
Metropolitan	635	15.4	15.3	8.5	11.0	6.9	7.0
Others	1393	14.5	12.5	7.8	9.1	6.7	6.6
**Types of main treatment institution**							
General hospital	2445	16.9	14.7	9.1	10.4	7.8	7.8
Hospital	235	8.7	7.8	4.5	5.8	4.4	3.9
Long-term care hospital	278	13.5	9.5	8.2	8.2	5.3	2.7
Clinic	125	3.5	7.4	2.2	4.8	1.3	2.9
**LOS**^**b**^		96.4	64.4	57.6	50.2	38.7	25.1
**Total**	**3083**	**15.5**	**14.0**	**8.4**	**9.9**	**7.1**	**7.3**

^a^*KRW* Korean Won, *NHI* National Health Insurance

^b^ The mean and standard deviation value of LOS

The association between end-of-life healthcare expenditures and the economic status of cancer patients are exhibited in Table 2. Compared to individuals in the ‘middle’ economic status group, those in the ‘high’ economic status group (RR 1.095, 95% CI 1.044–1.149) were likely to spend higher amounts. The trends found remained robust when analyzing healthcare expenditures separately for the periods of 12 to 4 months before death and 3 to 0 months before death.

**Table 2:** The association between end-of-life healthcare expenditures and economic status in cancer patients

Variables	Healthcare expenditures											
	Total				12 ~ 4 Months				3 Months ~ Death			
	RR	95% CI		***P***-value	RR	95% CI		***P***-value	RR	95% CI		***P***-value
**Economic status**												
Low	1.019	0.954	1.090	0.5700	0.987	0.906	1.076	0.7657	1.070	0.985	1.162	0.1097
Mid	1.000	–	–	–	1.000	–	–	–	1.000	–	–	–
High	1.095	1.044	1.149	0.0002	1.098	1.027	1.174	0.0059	1.123	1.051	1.199	0.0006
**Sex**												
Male	1.000	–	–	–	1.000	–	–	–	1.000	–	–	–
Female	0.953	0.910	0.998	0.0386	0.954	0.899	1.013	0.1219	0.960	0.899	1.025	0.2257
**Age (years)**												
~ 69	1.452	1.321	1.596	<.0001	1.849	1.617	2.115	<.0001	1.214	1.072	1.375	0.0023
70 ~ 74	1.318	1.245	1.395	<.0001	1.545	1.433	1.665	<.0001	1.174	1.089	1.266	<.0001
75 ~ 79	1.166	1.104	1.232	<.0001	1.260	1.172	1.356	<.0001	1.067	0.988	1.152	0.0965
80~	1.000	–	–	–	1.000	–	–	–	1.000	–	–	–
**Type of insurance coverage**^a^												
NHI (self-employed)	1.030	0.981	1.081	0.2306	1.069	1.000	1.143	0.0509	1.016	0.956	1.079	0.6171
NHI (employees)	1.000	–	–	–	1.000	–	–	–	1.000	–	–	–
**Types of cancer which diagnosed**												
Gastric cancer	1.000	–	–	–	1.000	–	–	–	1.000	–	–	–
Colorectal cancer	0.952	0.897	1.011	0.1060	1.058	0.981	1.142	0.1450	0.919	0.844	1.001	0.0536
Hepatocellular carcinoma	1.027	0.964	1.094	0.4110	1.151	1.060	1.250	0.0008	1.004	0.916	1.101	0.9293
Lung cancer	1.073	1.012	1.139	0.0190	1.273	1.178	1.376	<.0001	0.978	0.903	1.059	0.5827
**Survival time (from first diagnosis)**												
1y ~ 2y	1.000	–	–	–	1.000	–	–	–	1.000	–	–	–
2y ~ 3y	1.006	0.953	1.062	0.8351	1.099	0.968	1.249	0.1456	0.832	0.732	0.944	0.0045
3y ~ 4y	0.966	0.905	1.030	0.2858	1.111	0.977	1.262	0.1078	0.828	0.732	0.937	0.0028
4y ~ 5y	1.042	0.967	1.123	0.2783	0.970	0.851	1.107	0.6536	0.838	0.730	0.961	0.0118
5y+	1.108	1.004	1.224	0.0422	1.050	0.912	1.209	0.4970	0.907	0.782	1.053	0.1999
**Charlson Comorbidity Index**												
~ 2	1.000	–	–	–	1.000	–	–	–	1.000	–	–	–
3 ~ 5	1.128	1.036	1.229	0.0058	1.013	0.910	1.128	0.8165	1.192	1.062	1.338	0.0028
6~	1.373	1.275	1.478	<.0001	1.480	1.350	1.622	<.0001	1.276	1.151	1.414	<.0001
**Residing area**												
Capital area	1.000	–	–	–	1.000	–	–	–	1.000	–	–	–
Metropolitan	1.148	1.092	1.207	<.0001	1.159	1.087	1.235	<.0001	1.167	1.089	1.250	<.0001
Others	0.989	0.936	1.045	0.6889	1.011	0.935	1.093	0.7882	1.009	0.937	1.087	0.8131
**Types of main treatment institution**												
General hospital	4.415	3.659	5.328	<.0001	3.672	3.107	4.340	<.0001	4.123	3.262	5.211	<.0001
Hospital	2.372	1.952	2.882	<.0001	1.861	1.533	2.261	<.0001	2.278	1.788	2.901	<.0001
Long-term care hospital	2.092	1.722	2.542	<.0001	1.694	1.400	2.048	<.0001	1.740	1.362	2.222	<.0001
Clinic	1.000	–	–	–	1.000	–	–	–	1.000	–	–	–
**LOS (per day)**	1.010	1.009	1.010	<.0001	1.018	1.017	1.018	<.0001	1.031	1.030	1.032	<.0001
**Timing at death (per year)**	1.009	0.998	1.021	0.1100	1.022	1.006	1.038	0.0083	1.007	0.992	1.022	0.3872

^a^*KRW* Korean Won, *NHI* National Health Insurance

**Fig. 2:**
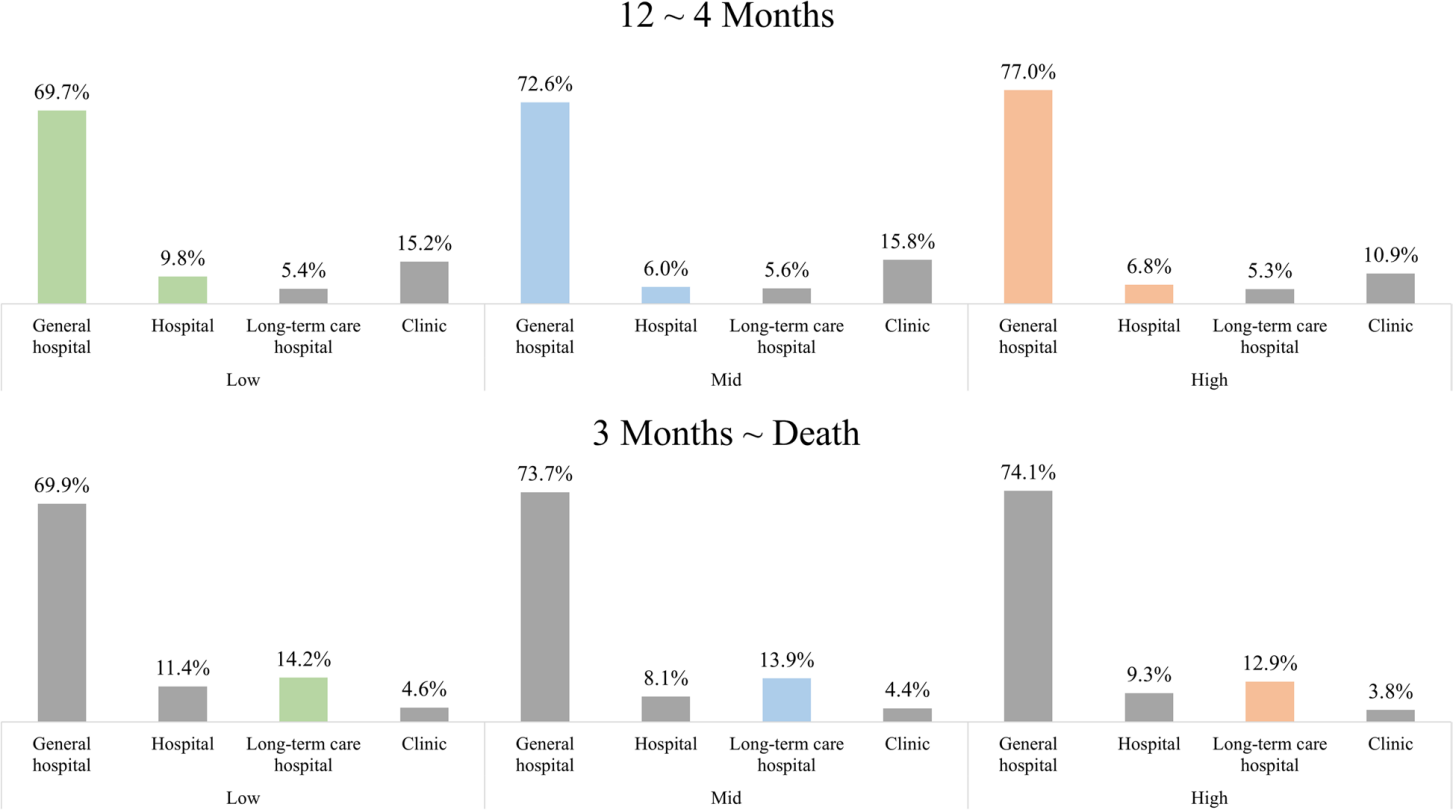
Type of medical institutions most visited according to the end-of-life period and economic status

Figure 2 reveals the type of healthcare institution most visited by cancer patients at end-of-life according to economic status. The percentage of individuals visiting a general hospital was highest in the ‘high’ economic status group (12–4 months: 77.0%, 3–0 months: 74.1%), followed by the ‘middle’ (12–4 months: 72.6%, 3–0 months: 73.7%) and ‘low’ (12–4 months: 69.7%, 3–0 months: 69.9%) economic status groups. In contrast, the percentage of individuals visiting a long-term care hospital was lower in the ‘high’ economic status group, particularly in the last 3 to 0 months of life (low: 14.2%; middle: 13.9%; high: 12.9%).

## Discussion

The findings reveal that differences in end-of-life healthcare costs exist according to economic status in Korean cancer patients. Expenditures increased in larger amounts in the last 3 months of life in all economic status groups. The higher economic status group tended to spend larger amounts on healthcare in their last year of life. The ratio between the ‘high’ and ‘low’ economic status group in terms of healthcare expenditures spent escalated slightly as participants neared end-of-life.

End-of-life healthcare costs for cancer merit attention because it is known to be particularly costly to cancer patients [19, 20]. The findings of this study show that end-of-life costs for cancer care tend to increase particularly in the last months of life, which is in general accordance with previous findings. For instance, a Canadian study concluded that end-of-life healthcare costs increase noticeably in the last 3 months of life [21]. Higher levels of cancer care expenditures were also found in a previous Korean study, which presented that expenditures increase in the last 3 months and peak at the last month before death [22]. Specifically, medical expenses at end-of-life medical were different according to income, which suggests that higher income households spend higher amounts on healthcare. Whilst other previous studies revealed similar patterns [23], others showed an inverse relationship between socioeconomic status and healthcare expenditures at end-of-life [24]. The findings are understandable considering that individuals belonging to lower socioeconomic commonly report worse health and functioning, which can lead to more frequent use of hospitalization services at end-of-life [25]. As no financial barriers exist to access services under the English National Health Service (NHS), differences in costs may be a reflection of poorer management rather than financial barriers [24].

Although there are many factors associated with hospitalization at end-of-life [26], out-of-hospital care is often used as a quality indicator for end-of-life-care [27]. Still, this may differ depending on the healthcare system of each country [1]. In the case of Korea, as most patients receive end-of-life care at hospitals, it is difficult to regard hospitalization care as lower quality end-of-life care [1]. At the same time, aggressive treatment at end-of-life care is increasing, which is known to be affected by various factors such as delays in conversion to palliative care or an increase in utilization of private health insurance [28]. As such, differences in the level of healthcare expenditures spend according to income level may be a reflection of differences in the ability to pay. Previous research has shown that highest willingness to pay for a quality-adjusted life year ranged $11,498 to $589,822 depending on the patient’s ability to pay [15]. This infers that while patients may be willing to pay for a better life, income level may be associated with their ability to pay for end-of-life care.

Due to the introduction of different healthcare policies on cancer, the co-payment rate for cancer care has reduced and is lower compared to other diseases. However, income level is still associated with the level of aggressiveness for cancer treatment. In this study, a higher proportion of patients in the ‘high’ economic status received treatment in general hospitals while a greater proportion of individuals is the ‘low’ economic status patients received treatment in long-term care hospitals at end-of-life. Long-term care hospitals focus on providing medical care and functional rehabilitation to a wide range of patients, including patients with geriatric or chronic diseases or undergoing recovery [29, 30]. Contrastingly, general hospitals tend to provide more innovative or high-cost services. As a result, clinical trials are also often concentrated in larger hospitals located in the metropolitan area in Korea [31]. Hence, the fact that more cancer patients belonging to higher economic status groups receive services in general hospitals may partially explain the differences in end-of-life healthcare expenditures found between income groups. However, it is not clear whether these patterns of healthcare expenditures reflect excessive or insufficient treatment in different income groups. Further research that considers the various factors that may impact the level of healthcare costs for end-of-life care according to income is needed.

This study has some limitations. First, only total costs for reimbursed healthcare services were incorporated in this study due to data limitation. Various costs, including transport or caregiving costs, could also not be considered. Actual levels of expenditure may also have been higher because this study did not consider uninsured costs for cancer treatment. Second, economic status was classified based on the level of NHI premium, which is a proxy for income. The level of NHI premium paid by an individual depends on the level of salary for employees and a variety of factors, such as assets, for the self-employed. Third, cancer stage was not considered due to data limitations. Fourth, as this study included only stomach, lung, colorectal, and liver patients, further research on this topic is needed for other cancer types. To partially overcome this limitation, the study only included first diagnosed patients who did not die within 1 year of diagnosis. Last, information on smoking and alcohol consumption were not available. However, the findings of this study are unique and relevant to policy makers because it is the first to analyze end-of-life healthcare costs in Korean cancer patients. Considering that end-of-life costs for cancer care constitute a large proportion of total healthcare costs, this study offers insight by revealing that expenditures tend to increase noticeably in the last 3 months of life and that differences exist in the amount spent according economic status.

## Conclusion

End-of-life healthcare costs for Korean patients with gastric, colorectal, lung, and liver cancer increase the most in the last months of life. End-of-life costs for cancer care were also greater in patients belonging to the higher economic status group after. Such tendencies may be associated with the fact that the higher economic status group tend to use general hospitals more. This study offers insight by showing that expenditures for cancer care tend to increase noticeably in the last 3 months of life and that differences exist in the amount spent according economic status.

## Supplementary Information

**Additional file 1.**

## Data Availability

Data is accessible from NHIS on request (https://nhiss.nhis.or.kr/bd/ab/bdaba000eng.do;jsessionid=7CiNGhrNuVY21GG5EaTQ9Gpi2krPhcmkIOw3I08spE9aF4c5pT4gVWIiuvwwuLUE.primrose22_servlet_engine10).
