# Supplementary material for: Disparities in healthcare expenditures according to economic status in cancer patients undergoing end-of-life care
Source: BMC Cancer. 2022 Mar 22;22:303. doi: 10.1186/s12885-022-09373-y (PMC8939210; doi:10.1186/s12885-022-09373-y)
Supplement: Supplementary file 1 — Additional file 1. [file 12885_2022_9373_MOESM1_ESM.docx]

**Supplemenatary Figure a.**

*South Korea's health care system operates as a single payment system. The entire populations is covered by the National Health Insurance (NHI).* *Insurance premiums are calculated based on the insured's level of income, such as salary, and also assets. Out-of-pocket expenditures vary according to hospitalization or outpatient care.* *Typically, inpatients pay 20% of total costs and outpatients pay 30-60% of total costs depending on the type of hospital visited. Low-income individuals or the disabled are separately covered by Medical Aid and such individuals can use medical services at a copayment level of 0-10%.* *The healthcare system consists of primary, secondary, and tertiary medical institutions and patients can generally choose a hospital to visit relatively freely.*

*Cancer is a high burden disease and Korea has expanded coverage for some high burden diseases. Since 2005, the government has gradually expanded coverage for cancer treatment and patients pay only 5% of total costs regardless of outpatient or inpatient care.* *There is no hospitalization fee for Medical-aid beneficiaries and the level of copayment for outpatient services is 0-5%.*

*
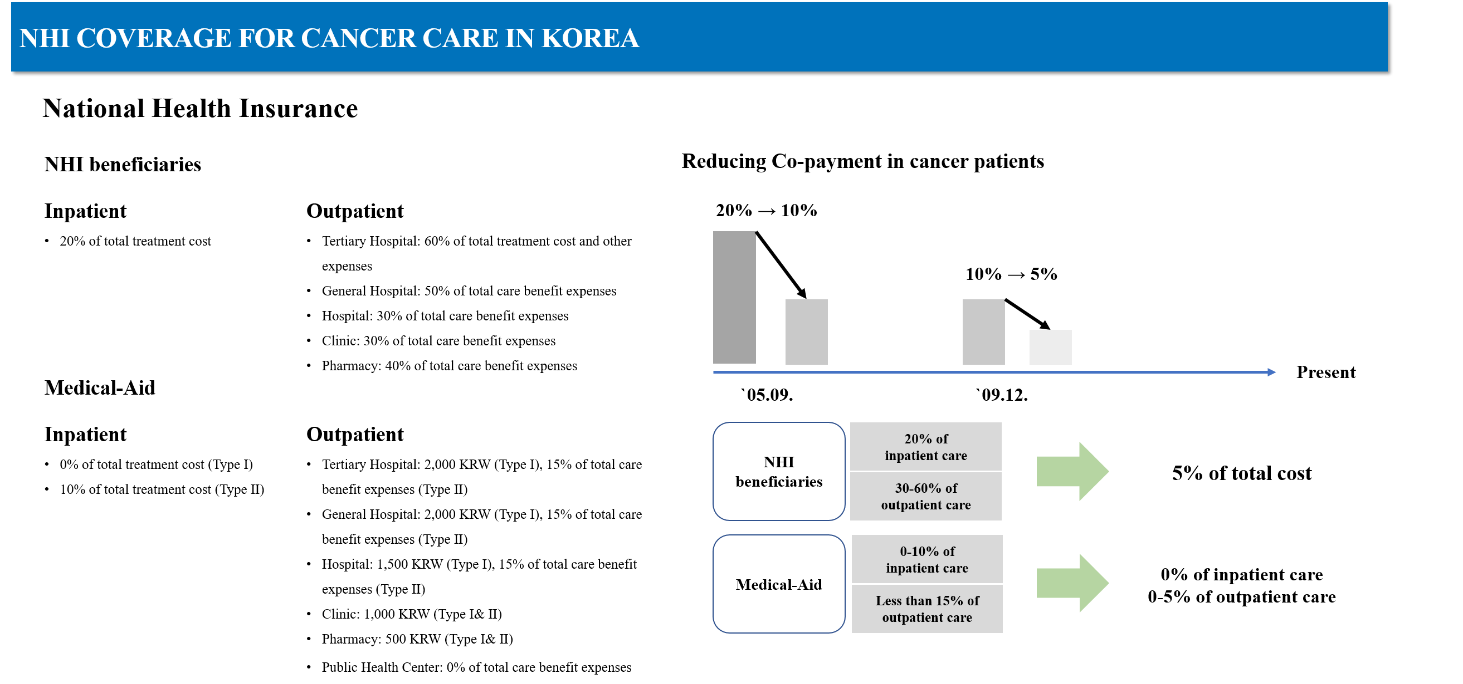
*

**Supplemenatary Figure a. NHI coverage for cancer care in Korea**
